# Tombusvirus P19 RNA silencing suppressor (RSS) activity in mammalian cells correlates with charged amino acids that contribute to direct RNA-binding

**DOI:** 10.1186/2045-3701-2-41

**Published:** 2012-12-06

**Authors:** Xiang Liu, Laurent Houzet, Kuan-Teh Jeang

**Affiliations:** 1 Molecular Virology Section, Laboratory of Molecular Microbiology, National Institute of Allergy and Infectious Diseases, National Institutes of Health, Bethesda, MD 20892, USA

## Abstract

**Background:**

Tombusvirus P19 is a protein encoded by tomato bushy stunt virus and related tombusviruses. Earlier studies have demonstrated that P19 is an RNA silencing suppressor (RSS) in plant cells. However, it has not been systematically investigated how P19 suppresses RNA interference in various mammalian cell settings.

**Results:**

We have studied the RSS effect of P19 in mammalian cells, HEK293T, HeLa, and mouse embryonic fibroblasts. We have individually mutated 18 positively charged residues in P19 and found that 6 of these charged residues in P19 reduce its ability to suppress RNA interference. In each case, the reduction of silencing of RNA interference correlated with the reduced ability by these P19 mutants to bind siRNAs (small interfering RNAs).

**Conclusions:**

Our findings characterize a class of RNA-binding proteins that function as RSS moieties. We find a tight correlation between positively charged residues in P19 accounting for siRNA-binding and their RSS activity. Because P19’s activity is conserved in plant and animal cells, we conclude that its RSS function unlikely requires cell type-specific co-factors and likely arises from direct RNA-binding.

## Background

RNA interference (RNAi) is a mechanism of gene regulation that is conserved in a wide range of organisms, from plants to animals [1-3]. RNAi is also reported to function as an antiviral defense against viral infections [4-9]. To counteract host cell RNAi-mediated immunity, viruses have evolved a variety of countermeasures, one of which is to encode RNA silencing suppressor (RSS) proteins [10-14]. Many RSS proteins have been reported; they include tomato bushy stunt virus (TBSV) P19 protein, rice hoja blanca virus NS3 protein, vaccinia virus E3L, influenza A virus NS1 protein, the Ebola virus VP35 protein, HIV-1 Tat protein, amongst others [5,15-23]. Currently, it is incompletely understood how each of these RSS proteins works mechanistically.

One of the better characterized RSS is the P19 protein [13,16,24] encoded by TBSV and related tombusviruses [25]. An association between P19 and siRNAs has been demonstrated in infected plants [26]. The crystal structure of P19-siRNA complex reveals that a P19 homodimer tightly binds a single 21-nucleotide (nt) siRNA duplex in a positively charged surface cleft, but that this binding is progressively weaker for a siRNA of 23–26 nt in size and become even weaker for a 19 nt siRNA [26,27]. Two tryptophan residues (W39 and W42) in P19 act as calipers to precisely bracket both ends of the siRNA duplex with a 2-nt 3’ overhang. Mutation of these two tryptophan residues was shown to greatly reduce RNAi suppression in *N. benthamiana* plants due to decreased binding of siRNA [26]. Upon TBSV infection of *N. benthamiana* and *N. clevelandii*, P19 contributes to regulating the manifestation of symptomatic phenotypes, such as apical necrosis and subsequent death [28,29].

To understand the mechanism of action of RSS proteins, it is important to determine whether cell specific proteins provide co-factor functions in the suppression of RNAi activities. Indeed, there is discordant data in the literature that suggest P19 does [30] or does not [31] work effectively as an RSS in human cells, including variant results between human 293T versus HeLa cells. Of relevance, several RSS proteins expressed by animal viruses have been demonstrated to maintain RSS activity in plants. For example, influenza A virus NS1 protein suppresses RNA silencing in plant cells by binding siRNA [32], and the expression of HIV-1 Tat protein in *N. benthamiana* restores GFP fluorescence by inhibiting RNA silencing downstream of the maturation step of dsRNA duplexes [33]. Here, we have re-examined the expression of TBSV plant virus P19 RSS protein in animal cells to determine the requirements for its suppression of RNA interference. We have assessed the RSS activity of TBSV P19 employing quantitative luciferase assays in mammalian HEK293T cells, HeLa cells, and mouse embryonic fibroblasts (MEFs). In our study, we have individually mutated eighteen positively charged amino acid residues and have found six that are involved in RNA-binding. We have determined a strict correlation between those charged residues needed (not needed) for RNA-binding and their necessary (unnecessary) contribution to the RSS activity of P19.

## Results

### P19 suppresses shRNA- and siRNA- mediated RNAi silencing in mammalian cells

**Figure 1:**
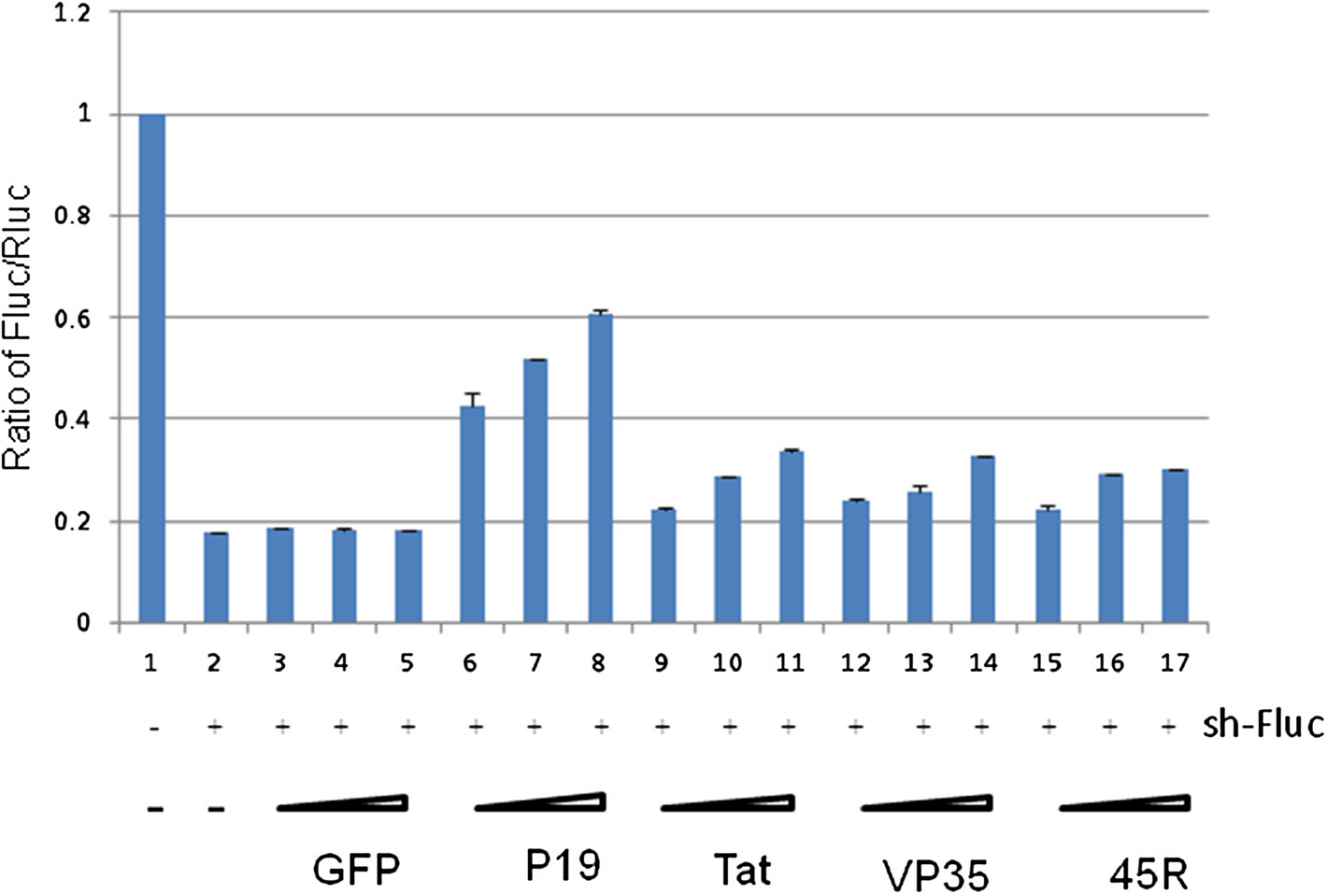
**shRNA-mediated RNAi silencing in HEK293T cells by P19, Tat, VP35 or 45R.** HEK293T cells were transfected with expression plasmids for firefly (Fluc) and Renilla luciferase (Rluc) together with a shRNA that targets Fluc (sh-Fluc, lanes 2–17) or a control irrelevant shRNA (shGFP, lane 1). As indicated, increasing amounts of expression plasmids for GFP, FLAG-tagged P19, Tat, VP35 or 45R (45 repeated arginines) were also co-transfected into HEK293T cells. Luciferase activities were quantified at 20 hours post transfection, and Fluc/Rluc ratios are graphed based on the averages from three independent experiments.

To investigate systematically TBSV P19 suppression of RNAi-silencing in mammalian cells, we first studied its activity in HEK293T cells, where its RSS activity, using a V5-epitope tagged P19 expression vector, was previously reported as inactive [31]. For this purpose, we co-transfected HEK293T cells with expression vectors for a Firefly luciferase (Fluc), a Renilla luciferase (Rluc), and a shRNA (small hairpin RNA) targeting Firefly luciferase mRNA (sh-Fluc) [34]. In this context, the expression of the shRNA, sh-Fluc, is expected to silence the Fluc mRNA while leaving undisturbed the Rluc mRNA. We also individually co-introduced into the transfected cells expression vectors for FLAG-tagged P19 (referred to hereafter simply as P19), HIV-1 Tat protein, VP35 Ebola virus protein, or a CMV-immediate early promoter driven expression vector that expresses a polypeptide of 45 repeated arginines (i.e. pCMV-45R). If the latter expression vectors produce RSS activity, we expect to measure a reduction in the ability of sh-Fluc to silence Fluc mRNA. After co-transfecting Fluc+Rluc+sh-Fluc with P19, Tat, VP35 or pCMV-45R into cells for 20 hours, the Fluc/Rluc ratios from individual HEK293T samples were determined by luminometric measurements (Figure 1). We observed that P19, Tat, VP35 and 45R (Figure 1) all provided dose-dependent RSS activities in HEK293T cells suppressing shRNA-mediated gene-silencing. In these assays, the RSS activity of P19 was slightly stronger than that shown by Tat, VP35 or the 45R peptide.

**Figure 2:**
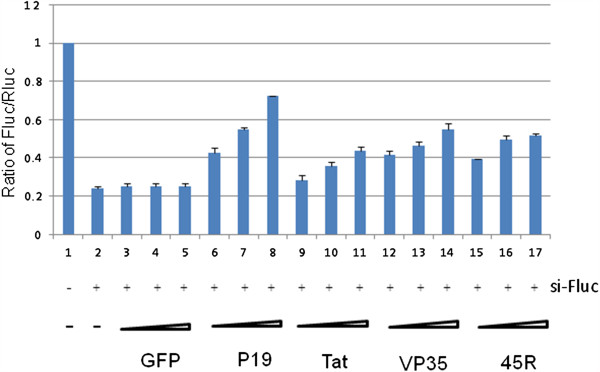
**Inibition of siRNA-mediated RNAi silencing in HEK293T cells by P19, Tat, VP35 or 45R.** HEK293T cells were transfected with expression plasmids for firefly (Fluc) and Renilla luciferase (Rluc) together with a siRNA that targets Fluc (si-Fluc, lanes 2–17) or a control scramble siRNA (lane 1). As indicated, increasing doses of expression plasmids for GFP, FLAG-tagged P19, Tat, VP35 or 45R (45 repeated arginines) were also transfected into HEK293T cells. Luciferase activities were quantified at 20 hours post transfection, and Fluc/Rluc ratios are graphed based on the averages from three independent experiments.

Because shRNA-mediated RNAi requires a Dicer processing step [35-37] while siRNA-mediated RNAi does not, we next asked whether similar findings would be achieved if we employed a siRNA (si-Fluc)-targeting Firefly luciferase mRNA in place of sh-Fluc. We thus compared the ability of P19, Tat, VP35 and 45R to suppress si-Fluc silencing of Fluc-mRNA. Figure 2 shows that P19, Tat, VP35, or 45R showed similar dose-dependent effects on si-Fluc (Figure 2) as they did on sh-Fluc (Figure 1).

### Point mutation of positively charged residues in P19 affects its suppression of RNA interference

**Figure 3:**
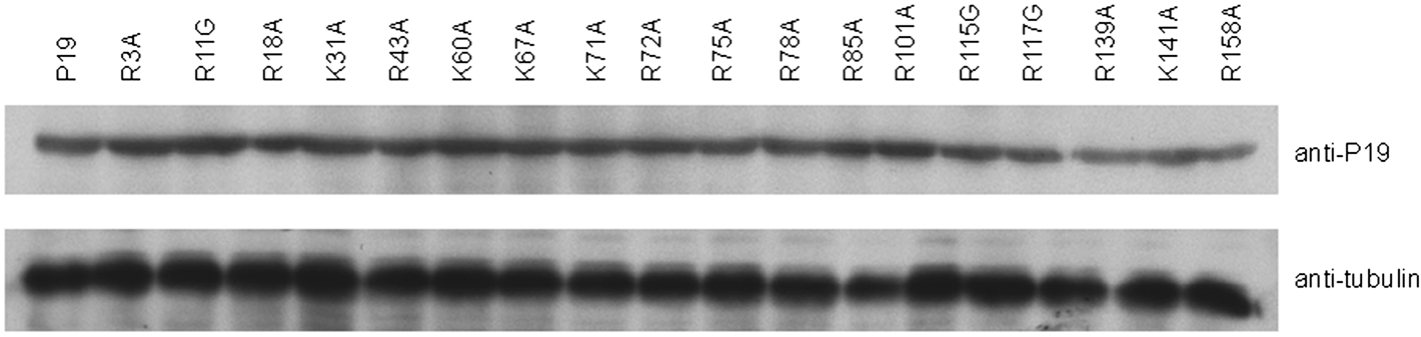
**Expression of P19 and mutants in HEK293T cells.** Wild type P19 or the indicated mutants were loaded in equal amounts and Western blotted using P19 specific polyclonal serum.

**Figure 4:**
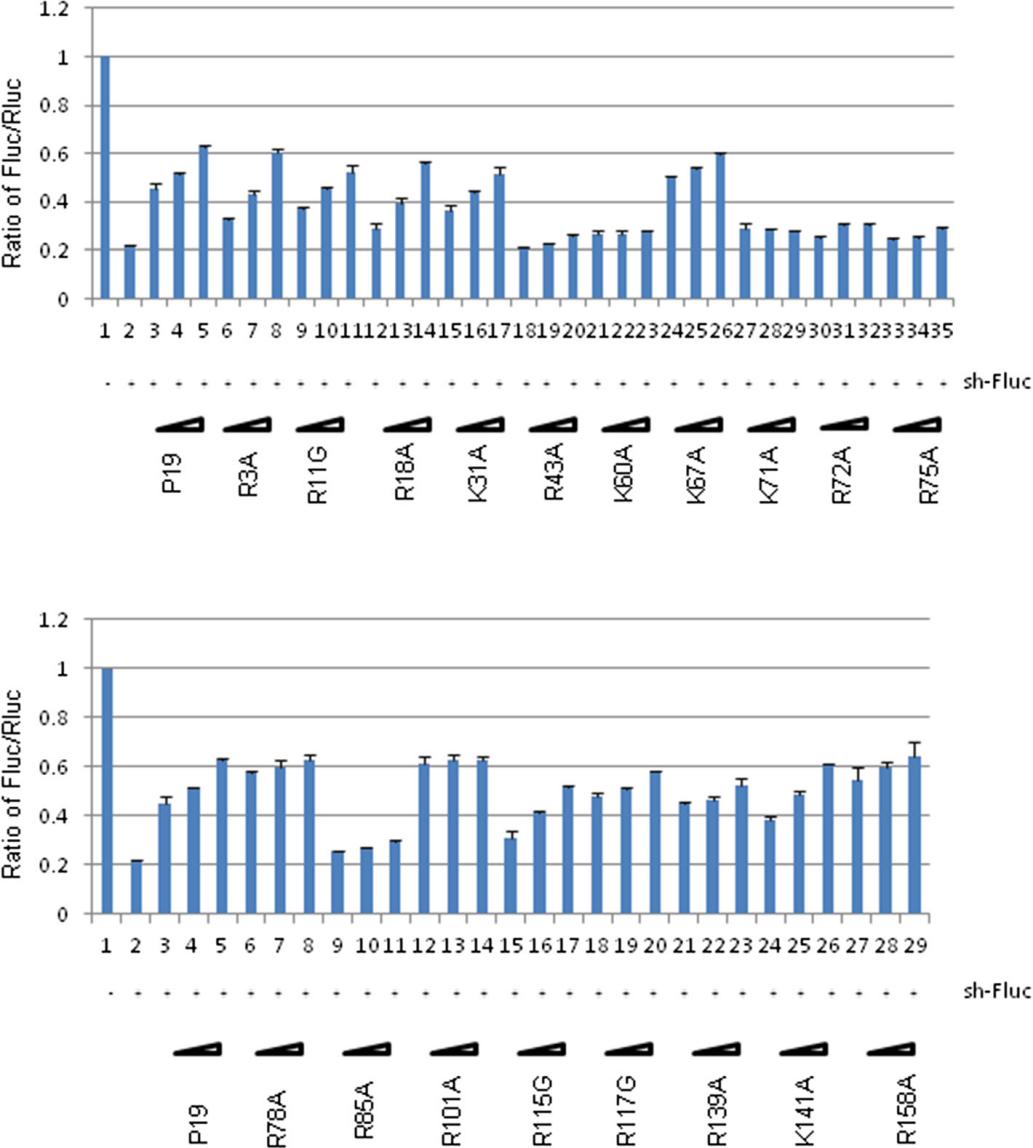
**Suppression of shRNA-mediated RNAi-silencing by P19 or P19 mutants in HEK293T cells.** HEK293T cells were transfected with expression plasmids for firefly (Fluc) and Renilla luciferase (Rluc) together with a shRNA that targets Fluc (sh-Fluc) or a control irrelevant shRNA (shGFP, lane 1). Increasing doses of the indicated expression plasmids for FLAG-P19 or FLAG-P19 mutants were also transfected into HEK293T cells. Luciferase activities were quantified at 20 h post transfection. Fluc/Rluc ratios are graphed based on the averages from four independent experiments. Please note that in the top and bottom graphs the identical values from wild type P19 are presented twice simply for the purpose of easier comparison with the values from the respectively graphed P19 mutants.

**Figure 5:**
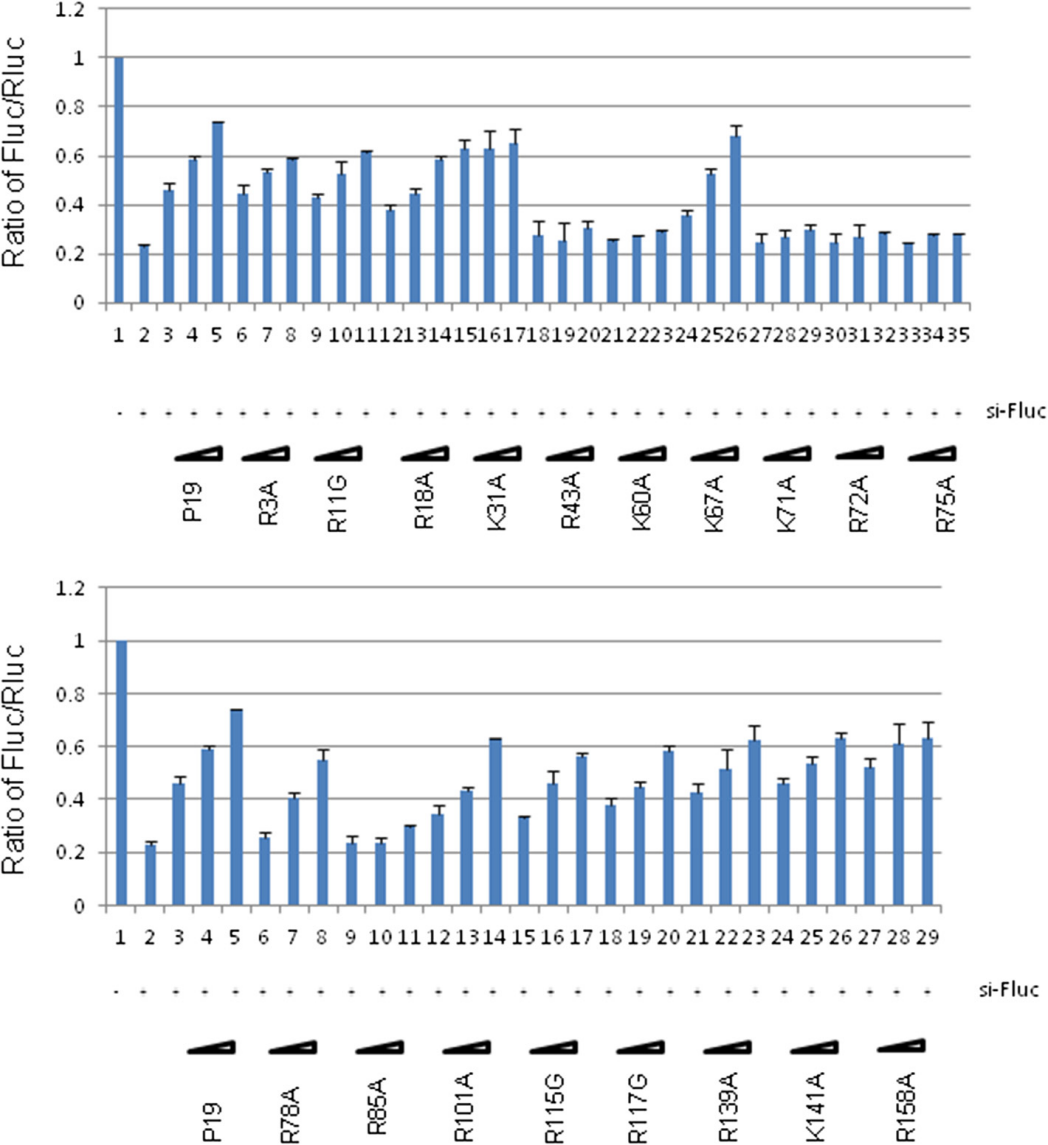
**Suppression of siRNA-mediated RNAi-silencing by P19 or P19 mutants in HEK293T cells.** HEK293T cells were transfected with expression plasmids for firefly (Fluc) and Renilla luciferase (Rluc) together with a siRNA that targets Fluc (si-Fluc) or a control irrelevant siRNA (scrambled siRNA, lane 1). Increasing doses of the indicated expression plasmids for FLAG-P19 or FLAG-P19 mutants were also transfected into HEK293T cells. Luciferase activities were quantified at 20 h post transfection. Fluc/Rluc ratios are graphed based on the averages from four independent experiments. Please note that in the top and bottom graphs the identical values from wild type P19 are presented twice simply for the purpose of easier comparison with the values from the respectively graphed P19 mutants.

Sequence analysis of TBSV P19 shows that there are 18 positively charged residues of either lysine or arginine that are considered generally important for binding nucleic acids. We individually point mutated all 18 positively charged residues to examine the impact of these changes on P19’s RSS activity. Western blotting of the expression of these point mutants demonstrated that none of the point changes significantly affected protein stability (Figure 3). In assays for RSS activity in HEK293T cells, we compared in parallel the wild type P19 and the 18 point mutants (Figure 4, 5). Amongst the 18 mutants that were tested, only 6 mutants (R43A, K60A, K71A, R72A, R75A and R85A) exhibited a loss of their RSS capability to suppress sh-Fluc-mediated RNAi silencing (Figure 4). Similar results were seen in RSS assays for the suppression of si-Fluc (Figure 5). Taken together, the results support that these 6 residues are of predominant importance to the RSS activity of P19 in mammalian cells.

### P19 residues contributory to RSS are critical for RNA-binding

**Figure 6:**
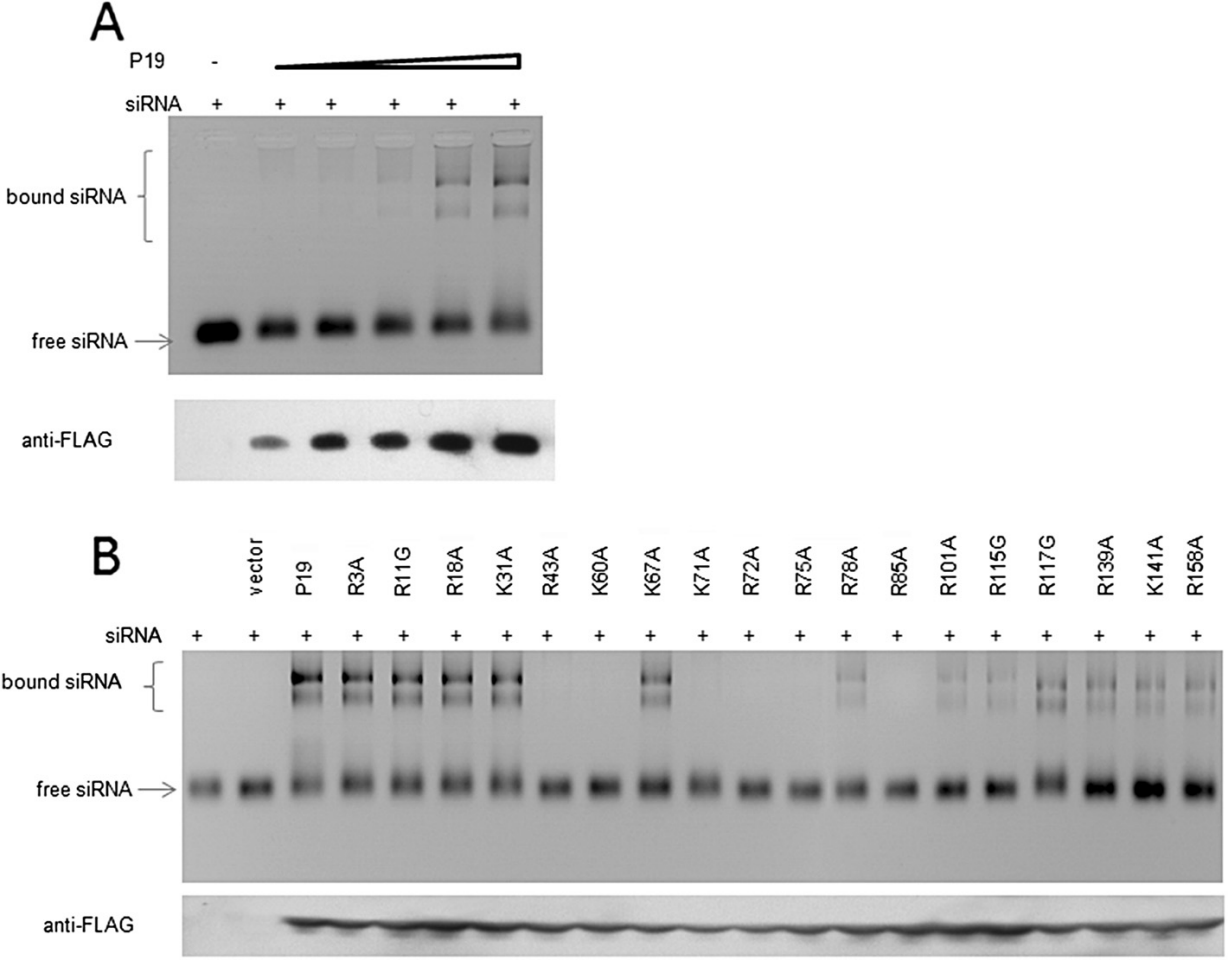
**In vitro RNA-binding by P19 and P19 mutants.** Gel shift assays were performed for FLAG-P19 and mutated FLAG-P19 proteins. **A)** Increasing amount of FLAG-P19 was incubated for 30 minutes with 0.2 μM si-Fluc at 25°C. The amount of FLAG-P19 were quantified by Western blot analysis using anti-FLAG antibody. **B)** Equal amounts of purified FLAG-P19 or mutants were incubated for 30 minutes with 0.2 μM si-Fluc at 25°C. RNA was separated on 2% TBE-agarose gel and visualized by staining with ethidium bromide. The protein expression of FLAG-P19 and mutants were shown using Western blot analysis.

Above, we found that individual changes in 6 positively charged residues in P19 produced a loss of RSS activity. One explanation for this loss of function could be that the amino acid changes affected the RNA-binding activity of P19. To address this possibility, RNA-binding native gel shift assays were carried out using si-Fluc RNA and purified P19 proteins. In titrating increasing amount of P19 in the presence of an excess of siRNA, we first established optimal binding condition of wild type P19 for siRNA (Figure 6A). Next, we checked the binding of P19 or its mutants to si-Fluc siRNA. Indeed, while wild type P19 and other mutants that did not affect RSS (e.g. R3A, R11G, R18A, K31A, K67A, R78A, R101A, R115G, R117G, R139A, R141A, R158A) bound si-Fluc siRNA, each of the six mutants (R43A, K60A, K71A, R72R, R75A, R85A) that lost RSS function failed to bind si-Fluc siRNA (Figure 6B). Collectively, these results support the interpretation that the RNA-binding activity of P19 in mammalian cells is the primary determinant of its RSS activity.

### PACT is not required for P19 suppression of sh-/si-RNA-mediated RNAi silencing in MEF cells

**Figure 7:**
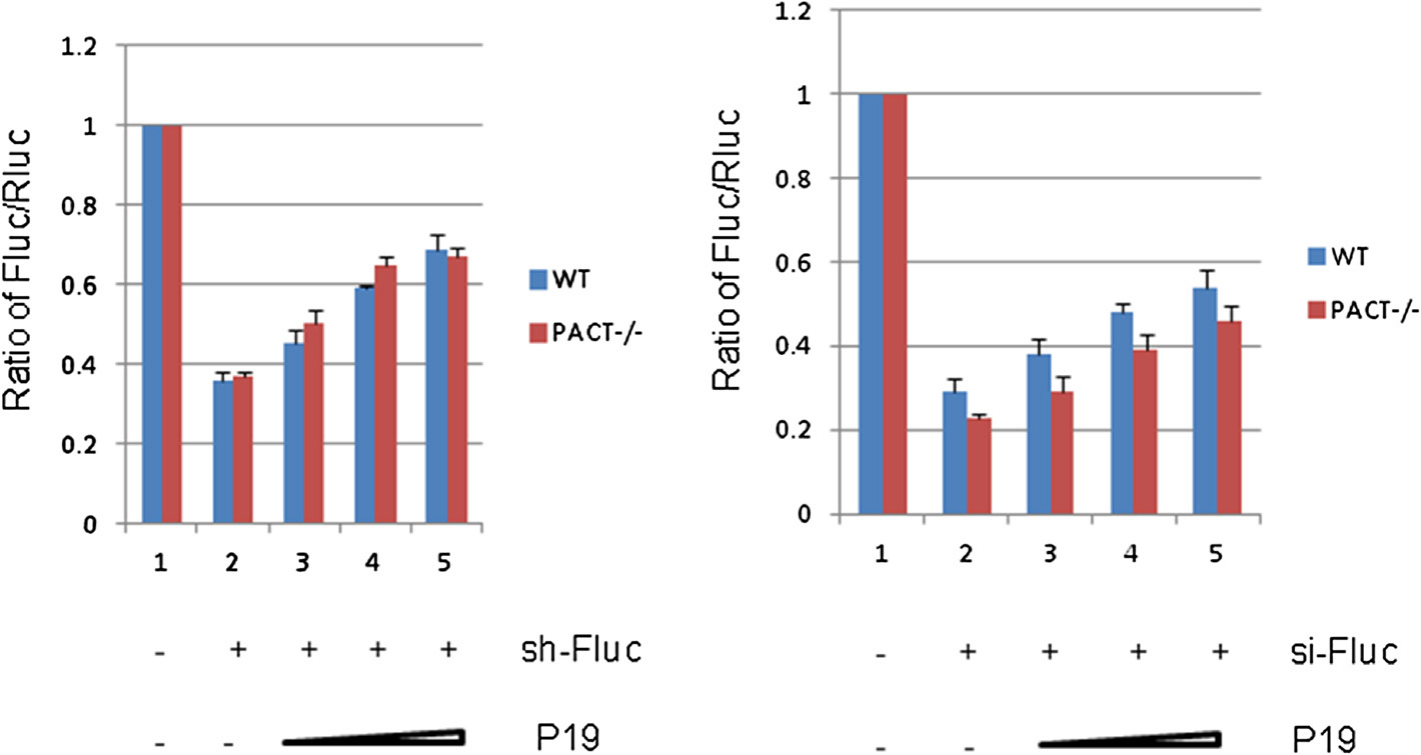
**P19 suppression of sh-RNA-mediated RNAi silencing does not require PACT.** WT and PACT^−/−^ MEFs were transfected with expression plasmids for firefly (Fluc) and Renilla luciferase (Rluc) together with sh-/si-RNAs that target Fluc (sh-/si-Fluc) or a control irrelevant shRNA (shGFP, lane 1, left panel) or scramble siRNA (lane 1, right panel). Increasing doses of expression plasmids for FLAG-P19 were also transfected into MEF cells. The results are averages from four independent experiments.

Previously, it was reported that untagged P19 exhibited RSS activity in HeLa cells [30] while epitope-tagged P19 had no RSS activity in 293T cells [31] Amongst various interpretations, one possibility was that perhaps P19’s RSS activity requires cell type-specific co-factor(s) that is present in HeLa, but not 293T cells. While our above results in 293T cells would indicate differently, to directly investigate cell type-specific influences on P19’s RSS, we extended our assays to primary mouse embryonic fibroblasts (MEFs). We tested P19’s RSS activity in wild type MEFs and also in MEFs that are knocked out for the *PACT* gene (i.e. PACT^−/−^) (Figure 7). We investigated the latter cells because the PACT protein has been reported to be an important component of the mammalian RNAi machinery [38,39]. In both wild type and PACT^−/−^ MEFs, P19 was effective in suppressing both sh- and si- RNA-mediated silencing of Fluc (Figure 7). These findings suggested that there is no cell type or species-specific differences between 293T cells and primary MEFs for P19’s RSS function and that this P19-activity does not require PACT as a co-factor.

**Figure 8:**
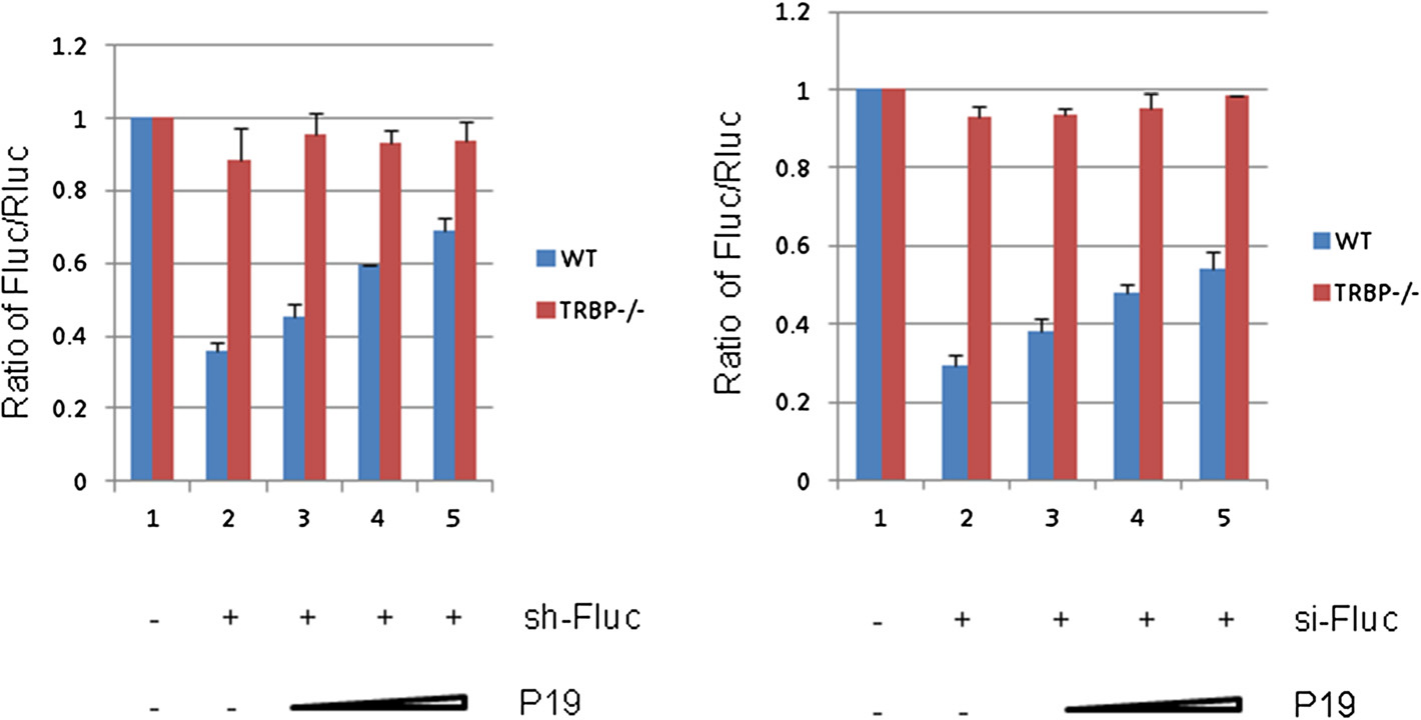
**TRBP is required for optimal sh-/si-RNA-mediated RNAi silencing.** WT and TRBP^−/−^ MEFs were transfected with expression plasmids for firefly (Fluc) and Renilla luciferase (Rluc) together with sh- /si-RNAs that target Fluc (sh-/si-Fluc) or a control irrelevant shRNA (shGFP, lane 1, left panel) or scrambled siRNA (lane 1, right panel). Increasing doses of expression plasmids for FLAG-P19 were also transfected into MEF cells. The results are averages from four independent experiments.

The PACT-related protein TRBP [40] is another cellular RNA-binding proteins that has been reported to be important for the loading of siRNAs into the RNA-induced silencing complex (RISC) for functional RNAi activity in mammalian cells [35,38,41-43]. We next checked if loss of TRBP would affect P19’s RSS activity. To address this requirement, we conducted sh-/si-Fluc mediated silencing of Fluc mRNA in wild type MEF and TRBP knock out MEF (i.e. TRBP^−/−^) and tested the functionality of P19’s RSS in these contexts (Figure 8). Although others had reported a functional redundancy and general equivalence between PACT and TRBP for mammalian RNAi function [44,45], in our assays, we found that sh-/si-Fluc-mediated silencing was very poor and was essentially non-functional in TRBP^−/−^ MEFs (Figure 8) at the same concentration of siRNA used in PACT^−/−^ MEFs . Thus in the context of our MEFs, our findings indicate a more important role of TRBP in siRNA loading into RISC that is not equivalently substituted by PACT [44,45]. Because si- and sh- RNA-mediated silencing of Fluc worked poorly in these TRBP-null MEFs, P19’s RSS activity in TRBP−/− MEFs could not be determined under these conditions. However, when higher siRNA concentrations were used in TRBP−/− MEFs, we could achieve RNAi-mediated silencing of Fluc mRNA that was suppressed by P19, suggesting that TRBP is also not a necessary co-factor for P19’s RSS in MEFs (data not shown).

## Discussion/conclusion

Here, we report that the RSS activity of FLAG-tagged TBSV P19 is conserved in human HEK293T cells and mouse embryonic fibroblasts (MEF). We also found that FLAG-P19 has similar RSS activity in HeLa cells (Additional file 1: Figure S1) as in HEK293T cells. Our work revisits earlier reports that untagged P19 exhibited RSS activity in HeLa cells [30] while epitope-tagged P19 showed little to no RSS activity in 293T cells [31], suggesting that neither epitope-tagging nor cell type-specific factors influence inherent P19 RSS activity in mammalian cells. We should, however, caution that our assay approaches are similar to, but not identical with, the previous studies; hence, we cannot exclude that small non-identical experimental details may account for the dissimilar findings.

In trying to characterize P19’s RSS activity in animal cells, we point mutated 18 positively charged lysine or arginine amino acids to neutral amino acid counterparts. In these analyses, we discovered 6 positively charged residues that are important for P19-mediated RSS effect. Mutation of these residues also abrogated the ability of the respective protein to bind siRNA. Our mutagenesis results on P19 in animal cells can be compared to parallel point mutation studies of P19 in plant cells. Thus, Chu *et al.* had shown that mutations upstream from residue K71 or downstream from residue R85 did not noticeably affect the ability of TBSV to systematically invade spinach plants [25]. Mutation of R72, R75 or R85 displayed a reduced lethal necrosis phenotype in three different plants (*N. benthamiana*, *N. clevelandii* and spinach) [25], and the mutation of R43 was shown to decrease the stability of interaction between P19 (R43) protein and siRNA in *N. benthamiana*[46]. Crystal structure of P19 revealed that K71 and R115 form direct contacts with phosphate groups in the siRNA [27]. Viewed in the above context, our results in mammalian cells show that K71 is important for RSS activity of P19, but mutation of R115 did not affect this activity. Previously, mutation of K60 in infected plants showed necrotic lower leaves, but not systemic collapse [25]; and our results also showed that mutation of K60A greatly reduced the RSS effect and RNA binding activity of P19. Therefore, for the most part, those positively charged residues that contribute to RSS activity of P19 maintain similar functional roles in mammalian and plant cells, further supporting the notion that the P19 RSS effect in plants and animals arises from co-factor independent direct RNA-binding.

An unexpected observation from our work is that sh-/si-Fluc-mediated silencing was more efficient in PACT^−/−^ MEFs (Figure 7) than TRBP^−/−^ MEFs (Figure 8). These results suggest a role for TRBP in siRNA loading into RISC that may not be equivalently substituted by PACT. Although both TRBP and PACT are found in the 500 kDa complex with Dicer and Ago2 and contribute to the processing of miRNA and shRNA, increasingly nuanced studies had indicated that TRBP appears to have a more critical role than PACT [44,45] in the cellular RNAi process. Indeed, a recent study showed that TRBP, but not PACT, can directly influence the specificity of Dicer cleavage of pre-miRNA [47]. Relevant to P19, our results suggest that neither PACT nor TRBP plays an essential co-factor role for P19’s RSS activity.

Our results here reinforce the earlier notion that many RNA-binding proteins can function as RNAi-suppressors [5,19,20,48,49] . Indeed, we have previously shown [34], and reaffirmed in Figure 1, that simple RNA-binding polypeptides like poly-arginine can exhibit RNAi-suppressing activity. Viral RNA-binding proteins like HIV-1 Tat and HTLV-1 Rex have evolved to serve virus-specific roles, but consistent with our current findings on P19, they also show RSS activity [20,50], suggesting that they participate in aspects of virus-cellular RNAi engagement [6]. RNAi activity contributes wide-ranging and diverse roles in cellular proliferation, gene regulation, development, metabolism, immune response, infection, and pathogenesis [51-54]. Physiologically, a reasonable notion is that organisms should have evolved biological means that either enhance or repress RNAi activities. We hypothesize that many cellular RNA-binding proteins [55] may possess suppressive RSS activities while others like TRBP may positively enhance RNAi function. Recently, a computational strategy was used to screen for small molecules with the potential to inhibit miRNA functions [56]. Going forward, further work on the discovery of small molecule inhibitors may help us develop tools to understand better how cellular RNA-binding proteins influence RNAi functions in cells.

## Methods

### Plasmids and reagents

The expression vectors for Fluc and Rluc are the pGL3-plasmid (Promega, Madison, WI) and the pRL-TK plasmid (Promega), respectively. pRS-shLuc (sh-Fluc), pRS-shGFP and pRS control plasmids were purchased from Origene. pCMVp19FL9 (FLAG-tagged), mammalian expression vector for P19, was a gift from Dr. Kathleen Boris-Lawrie (Ohio State University, USA). The plasmid of pcDNA3.1-VP35 was a gift from Dr. Stuart Nichols (Center for Disease Control, USA). The plasmids of pcDNA-TRBP and pCMV-45R (expression vector for 45 repeated arginines) were constructed in our laboratory. TBSV P19 rabbit polyclonal antibody was a kind gift from Dr. Herman B. Scholthof (Texas AM University, USA). siRNA to Firefly luciferase was from Invitrogen.

### Cell culture and transfection

HEK293T cells, wild type MEF and knockout MEF cells were maintained in Dulbecco’s modified Eagle’s medium (DMEM) supplemented with 10% fetal bovine serum (FBS), 2 mM/l L-glutamine and antibiotics in 5% CO_2_ at 37°C. Cells were transfected with 50 ng Fluc, 50 ng sh-Fluc (or sh-GFP), 5 ng Rluc, together with increasing dose (25 ng, 50 ng and 100 ng) of RSS proteins or P19 mutants. 25 pM si-Fluc siRNA were used to transfect both HEK293T cells and MEFs. PACT^−/−^ MEF cells are gifts from Dr. Ganes C. Sen (Cleveland Clinic, USA) [57,58]. TRBP^−/−^ mice are gifts from Dr. Robert E. Braun (Jackson Laboratory, USA) [59]. TRBP^−/−^ MEF cells were generated in our laboratory.

### Luciferase assay

Luciferase activity was quantified using the Dual-Glo Luciferase assay system (Promega) according to the manufacturer’s protocol.

### Western blot

The cells were washed with PBS twice and then lysed in lysis buffer (50 mM Tris–HCl, pH 7.5, 150 mM NaCl, 5 mM EDTA, 1% Triton X-100, 0.1% SDS) supplemented with protease inhibitor cocktail (Roche). The lysates were resolved by 12% SDS-PAGE and transferred to polyvinylidene fluoride membranes (Millipore). The membrane was incubated with primary antibodies, followed by alkaline phosphatase-conjugated secondary antibodies (Sigma-Aldrich). Signals were visualized using chemiluminescence following the manufacturer’s protocol (Chemicon).

### Immunoprecipitation

HEK293T cells were lysed with RIPA buffer for 20 minutes at 4°C. Lysates were clarified at 12,000 rpm for 10 minutes at 4°C, then incubated with anti-Flag beads (Sigma), and then rotated slowly at 4°C overnight. The antibody-bound complexes were washed three times and eluted by resuspending the beads with Flag-polypeptides. The supernatant was centrifuged and concentrated.

### Gel shift assay

Gel shift assay was performed using purified P19 or its mutants with 0.2 μM siRNAs in buffer containing 50 mM Tris–HCl (pH 7.4) and 100 mM NaCl. After incubation for 30 minutes at 25°C, the reaction mixtures were separated on 2% Tris-Borate-EDTA (TBE)-agarose gels. The RNA was visualized by staining with ethidium bromide at 1 μg/ml.

## Competing interests

The authors declare that they have no competing interests.

## Authors’ contributions

KTJ conceived of the study, and XL performed all the experiments in this paper. LH helped with the mouse embryo fibroblast studies. All authors analyzed data and wrote the manuscript. All authors read and approved the final manuscript.

## Supplementary Material

Additional file 1 **Figure S1.** sh-/si-RNA-mediated RNAi silencing in HeLa cells by P19, Tat, VP35 or 45R. **A**) Inhibition of shRNA-mediated RNAi silencing in HeLa cells by FLAG-P19, Tat, VP35 or 45R. HeLa cells were transfected with expression plasmids for firefly (Fluc) and Renilla luciferase (Rluc) together with a shRNA that targets Fluc (sh-Fluc, lanes 2–17) or a control irrelevant shRNA (shGFP, lane 1). As indicated, increasing amounts of expression plasmids for GFP, FLAG-P19, Tat, VP35 or 45R (45 repeated arginines) were also co-transfected into HeLa cells. **B**) Inhibition of siRNA-mediated RNAi silencing in HeLa cells by FLAG-P19, Tat, VP35 or 45R. HeLa cells were transfected with expression plasmids for firefly (Fluc) and Renilla luciferase (Rluc) together with a siRNA that targets Fluc (si-Fluc, lanes 2–17) or a control scrambled siRNA (lane 1). As indicated, increasing doses of expression plasmids for GFP, FLAG-P19, Tat, VP35 or 45R (45 repeated arginines) were also transfected into HeLa cells. Luciferase activities were quantified at 20 hours post transfection, and Fluc/Rluc ratios are graphed based on the averages from three independent experiments. Click here for file
