# Supplementary figures and images for: Tombusvirus P19 RNA silencing suppressor (RSS) activity in mammalian cells correlates with charged amino acids that contribute to direct RNA-binding
Source: Cell Biosci. 2012 Dec 6;2:41. doi: 10.1186/2045-3701-2-41 (PMC3533911; doi:10.1186/2045-3701-2-41)

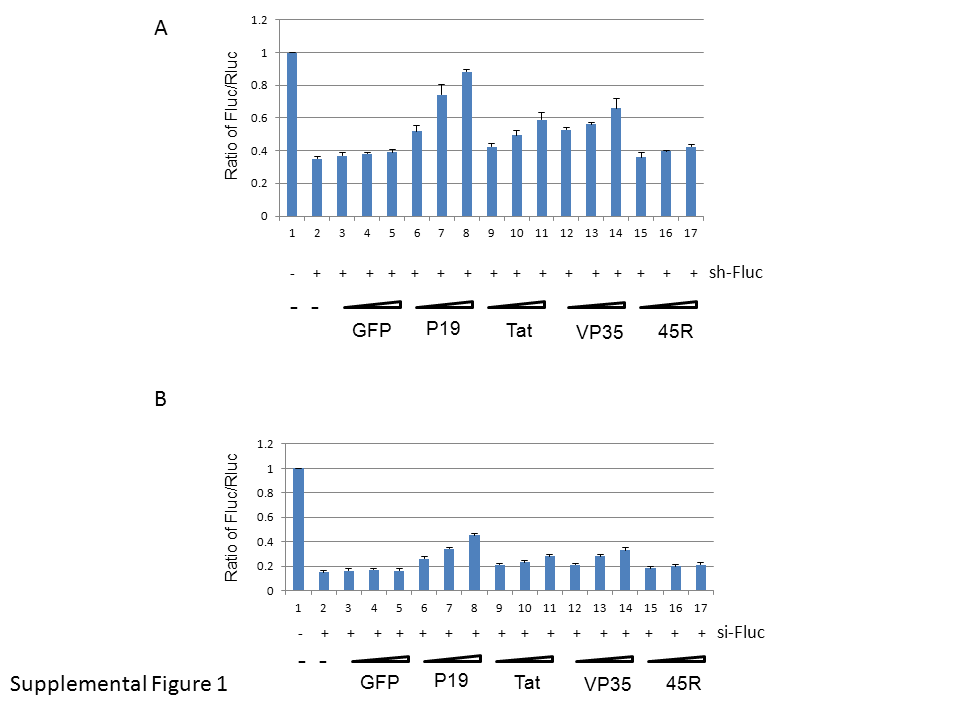

Supplement: Additional file 1 — Figure S1. sh-/si-RNA-mediated RNAi silencing in HeLa cells by P19, Tat, VP35 or 45R. A) Inhibition of shRNA-mediated RNAi silencing in HeLa cells by FLAG-P19, Tat, VP35 or 45R. HeLa cells were transfected with expression plasmids for firefly (Fluc) and Renilla luciferase (Rluc) together with a shRNA that targets Fluc (sh-Fluc, lanes 2–17) or a control irrelevant shRNA (shGFP, lane 1). As indicated, increasing amounts of expression plasmids for GFP, FLAG-P19, Tat, VP35 or 45R (45 repeated arginines) were also co-transfected into HeLa cells. B) Inhibition of siRNA-mediated RNAi silencing in HeLa cells by FLAG-P19, Tat, VP35 or 45R. HeLa cells were transfected with expression plasmids for firefly (Fluc) and Renilla luciferase (Rluc) together with a siRNA that targets Fluc (si-Fluc, lanes 2–17) or a control scrambled siRNA (lane 1). As indicated, increasing doses of expression plasmids for GFP, FLAG-P19, Tat, VP35 or 45R (45 repeated arginines) were also transfected into HeLa cells. Luciferase activities were quantified at 20 hours post transfection, and Fluc/Rluc ratios are graphed based on the averages from three independent experiments. [file 2045-3701-2-41-S1.tiff]
